# Structural insights reveal a recognition feature for tailoring hydrocarbon stapled-peptides against the eukaryotic translation initiation factor 4E protein†
†Electronic supplementary information (ESI) available. See DOI: 10.1039/c8sc03759k


**DOI:** 10.1039/c8sc03759k

**Published:** 2019-01-07

**Authors:** Dilraj Lama, Anne-Marie Liberatore, Yuri Frosi, Jessica Nakhle, Natia Tsomaia, Tarig Bashir, David P. Lane, Christopher J. Brown, Chandra S. Verma, Serge Auvin

**Affiliations:** a Bioinformatics Institute , A*STAR (Agency for Science, Technology and Research) , 30 Biopolis Street, #07-01 Matrix , Singapore 138671 . Email: chandra@bii.a-star.edu.sg ; Tel: +65 6478 8273; b Ipsen Innovation , 5, Avenue du Canada , Les Ulis , France 91940 . Email: sergeauvin@gmail.com ; Tel: +33 160 922481; c p53 Laboratory , A*STAR (Agency for Science, Technology and Research) , 8A Biomedical Grove, #06-04/05, Neuros/Immunos , Singapore 138648 . Email: cjbrown@p53lab.a-star.edu.sg ; Tel: +65 6478 8273; d Ipsen Bioscience , 650 East Kendall Street , Cambridge , MA 02142 , USA; e Department of Biological Sciences , National University of Singapore , 14 Science Drive 4 , Singapore 117543; f School of Biological Sciences , Nanyang Technological University , 50 Nanyang Drive , Singapore 637551

## Abstract

Stapled-peptides have emerged as an exciting class of molecules which can modulate protein–protein interactions. We have used a structure-guided approach to rationally develop a set of hydrocarbon stapled-peptides with high binding affinities and residence times against the oncogenic eukaryotic translation initiation factor 4E (eIF4E) protein. Crystal structures of these peptides in complex with eIF4E show that they form specific interactions with a region on the protein-binding interface of eIF4E which is distinct from the other well-established canonical interactions. This recognition element is a major molecular determinant underlying the improved binding kinetics of these peptides with eIF4E. The interactions were further exploited by designing features in the peptides to attenuate disorder and increase helicity which collectively resulted in the generation of a distinct class of hydrocarbon stapled-peptides targeting eIF4E. This study details new insights into the molecular basis of stapled-peptide: eIF4E interactions and their exploitation to enhance promising lead molecules for the development of stapled-peptide compounds for oncology.

## Abstract 2

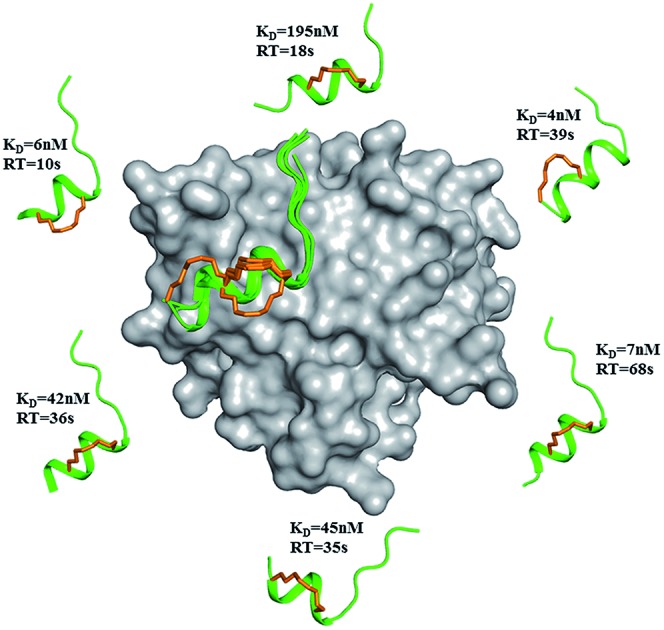
We have revealed a non-canonical recognition feature that can modulate the binding kinetics of hydrocarbon stapled-peptides interactions with the eIF4E protein.

## Introduction

The eukaryotic translation initiation factor 4E (eIF4E) protein is a vital component of the cap-dependent mechanism for mRNA translation.^1^ It binds the 7-methylguanosine (m^7^G) cap structure present at the 5′ end of mRNA molecules and recruits it as part of the eIF4F assembly which also includes two other partner proteins, eIF4A and eIF4G, to the translational machinery for protein synthesis.^1^,^2^ As eIF4E is the limiting component of the tripartite eIF4F complex, its expression levels and/or regulation are considered to be a critical determinant for mRNA translation.^3^ Specifically, in various types of cancers it has been observed that elevated levels of eIF4E selectively enhance the translation of malignancy-related mRNAs; in contrast, most cellular mRNAs can be efficiently translated at low concentrations of eIF4E.^2^ Thus, the therapeutic targeting of deregulated eIF4E in cancer has been suggested as an attractive clinical opportunity.^4^,^5^ In this regard, different strategies are being employed to develop compounds that can inhibit either (a) the phosphorylation of and/or phosphorylated eIF4E,^6^,^7^ (b) the recognition of mRNA by eIF4E,^8^ (c) the interaction of eIF4E with other proteins^9^ or (d) the translation of eIF4E mRNA itself,^10^ with varying degrees of success. The structural description of the interactions between eIF4E and peptides derived from its interacting protein partners such as eIF4G^11^ and 4EBP^12^ has provided an attractive opportunity to rationally design and develop peptidic inhibitors of eIF4E.

An emerging and exciting class of peptidic inhibitors are stapled-peptides, which have found increasing success in specifically targeting and inhibiting a wide range of protein–protein interactions (PPIs).^13^ Stapled-peptides are constrained (stapled) by chemical linkages, such as hydrocarbon chains, into a specific structural unit that mimics the conformation adopted by one of the epitopes in the protein–protein interaction. In addition, they generally exhibit improved metabolic stability and are better protected from proteolytic degradation compared to non-stapled peptides.^14^ Their clinical potential has been demonstrated by ALRN-6924, a first-in-class stapled-peptide candidate developed by Aileron Therapeutics that inhibits the p53: MDM2/MDMX interaction for treating advanced stage lymphoma.^15^ Hydrocarbon stapled-peptides were successfully used *in vivo* to disrupt interactions between pro- and anti-apoptotic members of the Bcl-2 protein family in order to modulate programmed cell death in cancer.^16^ They have also been explored as potential anti-viral compounds *in vitro* that can act by preventing protein-dimerization required for the correct assembly of virus capsids.^17^ Besides, they are suggested to have utility in modulating different pathways in numerous other pathologies highlighting the exciting promise held by this class of molecules as therapeutic solutions for several diseases.^18^ All together, these findings exemplify the utility of hydrocarbon stapled-peptides to form what is often termed “a third class of medicines” and thereby expand the druggable target space as PPI inhibitors.^19^

We have previously used structure-based rational design and optimization strategies to develop the first generation of hydrocarbon stapled-peptides against eIF4E.^20^ These are the only compounds currently reported in the literature that are able to bind to the protein-binding interface of eIF4E with nanomolar affinity. In contrast, small molecule inhibitors of the eIF4E/eIF4G interaction such as 4E1RCat^21^ and 4EGI-1 9 exhibit micromolar range affinity for eIF4E. Moreover, it was recently shown that 4EGI-1 binds to a non-canonical site on eIF4E and allosterically regulates the binding properties of peptides derived from eIF4G and 4EBP proteins.^22^ The weaker affinity of these small molecules likely originates from targeting an interface on eIF4E which is relatively flat lacking in deep grooves.^23^ Stapled-peptides may achieve higher affinities due to their large surface area for association and their canonical “YXXXXLφ” motif enabling specific strong interactions.^20^

In this study, we describe the rational design and development of highly potent second generation hydrocarbon stapled-peptides against eIF4E with enhanced binding kinetics and improved scaffold in terms of their degree of ordered helical character. We explore their modes of interaction with eIF4E through high resolution crystallographic data complemented by computational modeling which reveals new molecular insights into their mechanism of recognition. The work illustrates the generation of a distinct class of hydrocarbon stapled-peptides as potential lead compounds for drug development targeting eIF4E.

## Results

### Structure of 12mer stapled-peptide bound to eIF4E reveals an untapped patch

**Fig. 1:**
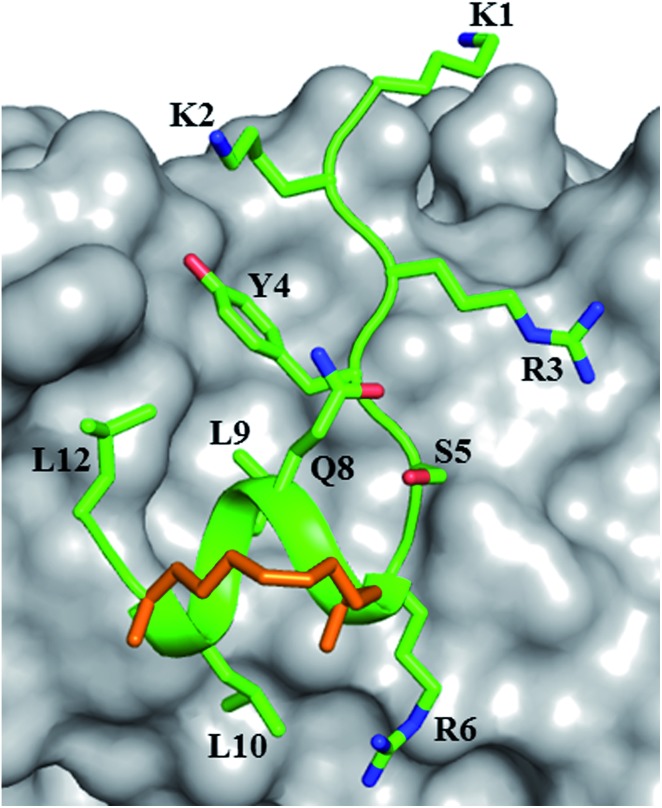
sTIP-04 peptide. Crystal structure of hydrocarbon stapled sTIP-04 peptide (^1^KKRYSR*QLL*L^12^) in complex with eIF4E (PDB ID: ; 4BEA). The protein eIF4E is shown in surface (gray) and the backbone of the peptide in ribbon (green) representations respectively. The side-chain of the peptide residues are explicitly shown in stick representation and labeled. The hydrocarbon linker is highlighted in orange color. This depiction is followed in the rest of the figures unless specified. All the molecular graphics figures were created using PyMol molecular visualization software (Schrödinger).

The previously optimized 12mer stapled-peptide (sTIP-04: ^1^KKRYSR*QLL*L^12^)^20^ (Fig. 1) was used as a template for the derivation of two different *i*, *i* + 4 hydrocarbon stapled-peptides: (i) (sTIP-05: ^1^KKRYSR*QLL*F^12^) with an L12F substitution at the C-terminus and, (ii) (sTIP-06: ^1^RIIYSR*QLL*L^12^) with an “RII^3^” substitution at the N-terminus, based on the 4EBP protein epitope.^12^ These derivatives were designed to incorporate different chemical features with the anticipation to induce favorable cellular activity. Both peptides were synthesized and their binding affinity for eIF4E was determined to be 6.6 nM and 0.8 nM for sTIP-05 and sTIP-06 respectively (Table 1), demonstrating that they were very good binders, similar to the parent sTIP-04 peptide which had an affinity of 5.0 nM.^20^ This result emphasized that the core of the 12mer stapled-peptide is well optimized and the terminal substitutions had limited impact on binding.

**Table 1:** Binding parameters of hydrocarbon stapled-peptides against eIF4E from surface plasmon resonance experiments

Peptide	Sequence^*a*^	*k*_on_ (M^–1^ s^–1^)	*k*_off_ (s^–1^)	*K*_D_ ^*b*^ (nM)	RT^*c*^ (s)
sTIP-05	Ac-KKRYSR*QLL*F-NH_2_	1.5 ± 0.3 × 10^7^	9.4 ± 0.8 × 10^–2^	6.6 ± 0.7	10.8 ± 0.9
sTIP-06	Ac-RIIYSR*QLL*L-NH_2_	2.7 ± 0.7 × 10^8^	2.2 ± 0.8 × 10^–1^	0.8 ± 0.1	6.0 ± 2.4
sTIP-07	Ac-KKRYSR*QLL*FW-NH_2_	6.5 ± 0.3 × 10^5^	2.8 ± 0.1 × 10^–2^	42.5 ± 0.5	36.3 ± 1.0
sTIP-08	Ac-RIIYSR*QLL*L&-NH_2_	6.2 ± 0.2 × 10^5^	2.8 ± 0.1 × 10^–2^	45.2 ± 0.6	35.5 ± 1.3
sTIP-09	Ac-KKRYSR*QLL*FRRR-NH_2_	2.2 ± 0.3 × 10^6^	1.6 ± 0.3 × 10^–2^	7.8 ± 1.9	68.8 ± 13.8
sTIP-10	Ac-KKRYSREQLL*FQR*-NH_2_	6.6 ± 0.1 × 10^6^	2.6 ± 0.5 × 10^–2^	4.3 ± 0.4	39.7 ± 3.1
sTIP-11	Ac-KRYSR*QLL*F-NH_2_	2.3 ± 0.9 × 10^7^	4.6 ± 1.2 × 10^–1^	21.9 ± 3.8	2.3 ± 0.6
sTIP-12	Ac-RYSR*QLL*F-NH_2_	4.2 ± 1.9 × 10^5^	1.4 ± 0.2 × 10^–1^	397.3 ± 131.1	7.1 ± 1.0
sTIP-13	Ac-YSR*QLL*F-NH_2_	NA	NA	NA	NA
sTIP-14	Ac-RYSR*QLL*LFR-NH_2_	3.4 ± 1.6 × 10^5^	5.8 ± 1.4 × 10^–2^	195.2 ± 49.7	18.3 ± 4.5
sTIP-15	Ac-RYSREQLL*FQR*-NH_2_	4.2 ± 2.3 × 10^6^	2.4 ± 1.5 × 10^–1^	54.8 ± 6.4	6.7 ± 4.1

^*a*^The sequence of the stapled-peptides synthesized with an acetylated (Ac) N-terminus and an amidated (NH_2_) C-terminus. The specific location where the non-natural amino acids are incorporated to form the hydrocarbon linker is indicated by “*”. & = Lys(ButPhI).

^*b*^Binding affinity (kinetic *K*_D_) measured as a ratio of “*k*_off_/*k*_on_”.

^*c*^Residence Time (RT) measured as “1/*k*_off_”. The values reported are mean ± SEM from at-least two independent experiments. The binding affinity estimated for sTIP-13 was in the micromolar range (4 μM to 36 μM). NA: not applicable. Also see Fig. S1 and S2 for sensogram data.

**Fig. 2:**
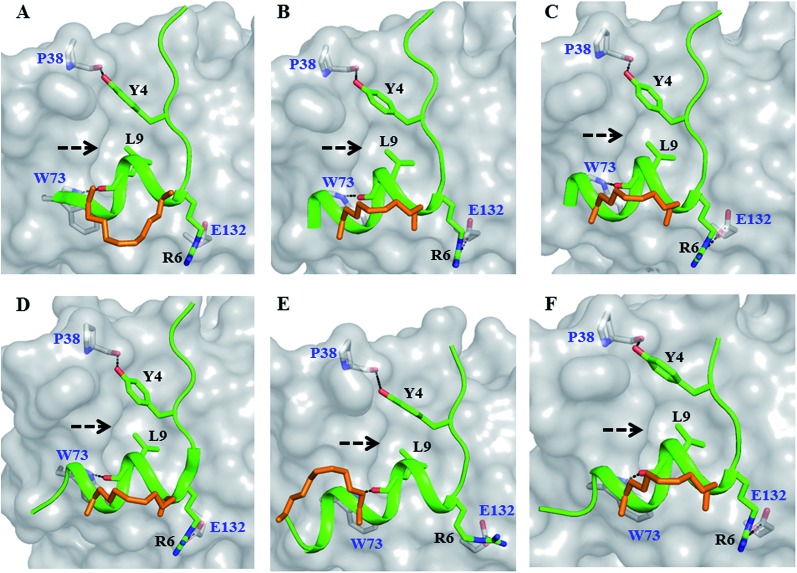
Canonical binding and interactions. Crystal structures of (A) sTIP-05 (PDB ID: 5ZJY), (B) sTIP-07 (PDB ID: ; 5ZJZ), (C) sTIP-08 (PDB ID: ; 5ZK9), (D) sTIP-09 (PDB ID: ; 5ZML), (E) sTIP-10 (PDB ID: ; 5ZK5) and (F) sTIP-14 (PDB ID: ; 5ZK7) hydrocarbon stapled-peptides in complex with eIF4E underlining the common binding mode and conserved interactions across all the structures. The backbone of the peptides is shown in ribbon (green), the protein in surface (gray) and the hydrocarbon linker is explicitly shown in stick (orange) representation. The backbone stereochemistry of the hydrocarbon linker in sTIP-05 is (*R*,*R*) whereas all the other peptides are in the (*S*,*S*) configuration. The residues involved in hydrogen-bond and salt-bridge interactions are indicated. The pocket where the conserved leucine residue (L9) docks onto the protein is specified by an arrow. The residue numbering for the protein is done as per the native eIF4E protein sequence (Uniprot ID: P06730).

**Fig. 3:**
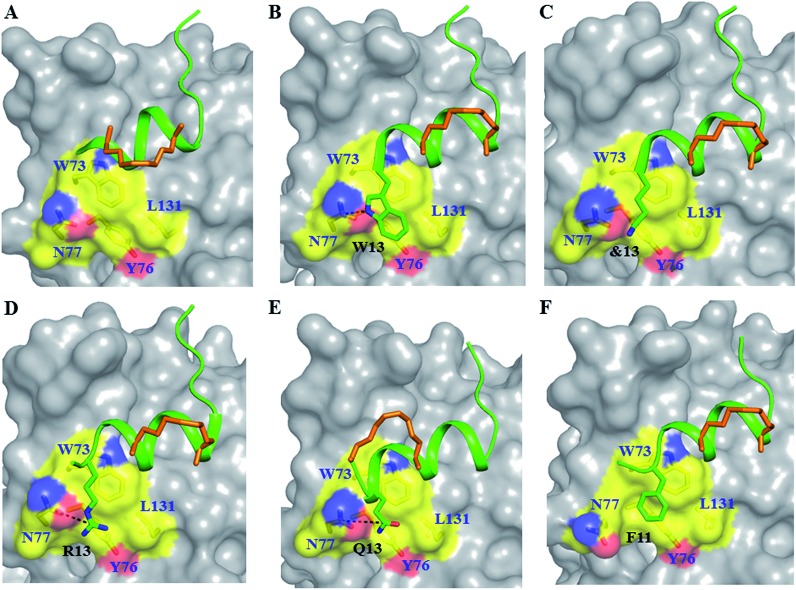
Untapped patch and its engagement. Crystal structures of (A) sTIP-05 (PDB ID: 5ZJY), (B) sTIP-07 (PDB ID: ; 5ZJZ), (C) sTIP-08 (PDB ID: ; 5ZK9), (D) sTIP-09 (PDB ID: ; 5ZML), (E) sTIP-10 (PDB ID: ; 5ZK5) and (F) sTIP-14 (PDB ID: ; 5ZK7) hydrocarbon stapled-peptides in complex with eIF4E highlighting the untapped patch on the protein and its engagement by different peptides. The residues forming the patch on eIF4E are emphasized with a different colour combination as compared to the rest of the protein. The side-chain of the residues from the peptide that interact with the patch are explicitly shown and the hydrogen-bond wherever formed is indicated. Residue “&13” is the resolved “Lys” moiety of the modified “Lys(ButPhI)” amino acid in sTIP-08.

The crystal structure of sTIP-05 in complex with eIF4E was resolved (Fig. S3A and Table S1†) which revealed that the peptide was bound with an N-terminal extended conformation and a regular helical structure towards the C-terminal, including the *i*, *i* + 4 staple (Fig. 2A). The hydrocarbon linker was found to be exposed to the solvent and did not engage with eIF4E. The specific interactions observed in other peptide: eIF4E structures,^11^,^12^ including a hydrogen-bond and salt-bridge between Y4-P38 and R6-E132 respectively, docking of the sidechain of L9 into a shallow pocket on eIF4E and a hydrogen-bond between the peptide backbone and the W73 side-chain of eIF4E, are all preserved in this crystal structure (Fig. 2A). In addition to these canonical interactions, we noticed an exposed untapped patch on the surface of eIF4E with potential to be targeted by interactions with the C-terminal end of a modified peptide (Fig. 3A). This region comprised of residues W73, Y76, N77 and L131, offering aromatic, hydrophobic and hydrogen-bonding properties. Next, molecular dynamics (MD) simulations were carried out on the crystallographic complex of sTIP-05: eIF4E and on a modeled complex of sTIP-06: eIF4E (generated by constructing *in silico* amino acid changes in the crystal structure of sTIP-05: eIF4E complex). They showed that the patch on eIF4E remained solvent exposed and neither of the 12mer peptides could engage it (Movies S1 and S2†). We hypothesized that a peptide extension at the C-termini may help engage this patch for more efficient target binding.

### Stapled-peptide derivatives engage the untapped patch on eIF4E for binding

To address the above hypothesis, we synthesized two 13mer stapled-peptide derivatives, sTIP-07 and sTIP-08, by extending the C-termini of sTIP-05 and sTIP-06 with a tryptophan and a modified amino acid “Lys(ButPhI)” respectively. These derivatives were made as the two additional residues have diverse physical characteristics besides possessing hydrophobic, aromatic and hydrogen-bonding features in their side-chains which collectively provide the essential physicochemical properties to examine the effectiveness of their potential interaction with the patch. Experimental *K*_D_ of both peptides was comparable to their respective parent molecules (Table 1). However, the binding kinetics (*k*_on_ and *k*_off_) were observed to be more sensitive to the change in the length of the peptide. The rate of association (*k*_on_) decreased appreciably for sTIP-07/sTIP-08 as compared to sTIP-05/sTIP-06 (Table 1). The lifetime of binding measured in terms of the residence time (RT = 1/*k*_off_) was also found to be higher for the 13mer peptides (∼36 seconds) compared to their 12mer counterparts (10 seconds for sTIP-05 and 6 seconds for sTIP-06). Crystal structures of the complexes of sTIP-07 and sTIP-08 with eIF4E were determined (Fig. S3B, C and Table S1†). The essential interactions made by the parent 12mer peptides were retained (Fig. 2B and C). More significantly, the added tryptophan (W13) in the sTIP-07 structure was observed to indeed efficiently interact with and engage the patch on eIF4E (Fig. 3B). W13 formed pi-stacking interactions with W73 and Y76 and hydrophobic interactions with L131. In addition, the amide nitrogen of the side-chain of W13 was also observed to form hydrogen-bond interaction with the side-chain of N77. In the crystal structure of sTIP-08 with eIF4E, only the density for the lysine moiety (Lys) of the modified residue “Lys(ButPhI)” (&13) was visible (Fig. 1C), forming hydrophobic interactions with W73, Y76 and L131 in the patch and potentially engaging the side-chain of N77 *via* a hydrogen-bond (Fig. 2C).

**Fig. 4:**
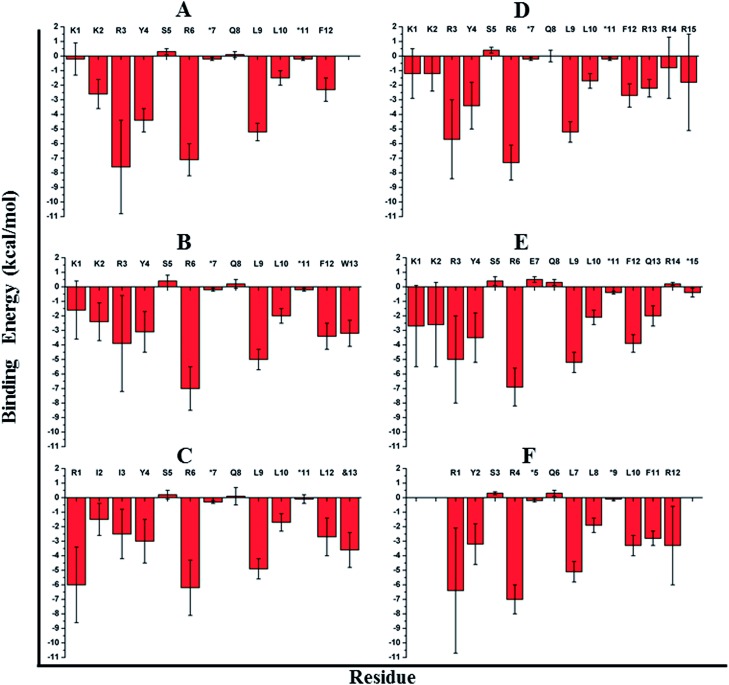
Residue-wise binding energy contribution. The average binding energy and standard deviation is computed from the ensemble of structures generated from the MD simulations of (A) sTIP-05, (B) sTIP-07, (C) sTIP-08, (D) sTIP-09, (E) sTIP-10 and (F) sTIP-14 hydrocarbon stapled-peptides in complex with eIF4E. The amino acid sequence of the respective peptides is indicated in the plot. The non-natural amino acids forming the hydrocarbon linker are represented by “*”. The calculation was done using the Molecular Mechanics/Generalized Born Surface Area (MM/GBSA) method by following the same procedure and parameters as described previously.^20^

We computationally modeled the missing functional group of &13 and subjected sTIP-07 and sTIP-08 complex structures to MD simulations. Analysis of the energies characterizing the simulations (including sTIP-05: eIF4E structure) showed that Y4, R6, L9, L10 and F12/L12 from the peptide made significant and stable energetic contributions (–1.7 to –7.2 kcal mol^–1^) to their binding with eIF4E consistently across all the three complexes (Fig. 4A–C). The hydrophobic/aromatic interactions between W13 and the residues from the protein as observed in the sTIP-07 crystal structure were fairly stable except for the W13–N77 hydrogen-bond (occupancy < 20%, Movie S3†), which nevertheless contributed to favorable binding energy (–3.2 kcal mol^–1^) from this residue (Fig. 4B). The “ButPhI” functional group of residue &13 in sTIP-08 was observed to be dynamic in the simulation and did not form any stable interactions with the protein (Movie S4†). Conversely, the “Lys” chain remained largely bound to the protein surface and significantly contributed to the binding energy (–3.6 kcal mol^–1^) between the protein and &13 at the C-terminus (Fig. 4C). This physical association with the protein could be the primary reason for the relative stability of the moiety and hence its resolution in the crystal structure. In summary, extending the C-termini of two stapled peptides with tryptophan or effectively lysine resulted in the efficient engagement of a previously untapped patch on eIF4E and an increase in the residence time of the peptide–protein complex.

### C-Terminal extension provides new stapling opportunity and enhances binding duration

We decided to further increase the length of the peptides towards the C-terminus with multiple residues, together with stapling around the extension to examine if the interaction with the patch can be further exploited. Two 15mer peptide derivatives of sTIP-05 (sTIP-09: ^1^KKRYSR*QLL*FRRR^15^ and sTIP-10: ^1^KKRYSREQLL*FQR*^15^) were synthesized, one with an “^13^RRR^15^” sequence extension and the other with an *i*, *i* + 4 hydrocarbon staple shifted to positions 11 and 15 of the peptide sequence which encapsulates the “^12^FQR^14^” sequence at the C-terminus. Arginine and glutamine represented another varied combination of residues with complementary chemical properties to examine against the patch and besides, arginine has been reported to improve peptide cellular permeability^24^ which should be useful in the further development of these peptides. In sTIP-10, position 7 was substituted by a glutamic acid (E7) as is seen in the sequence of the parent eIF4G1 peptide.^25^ Once again, the binding affinities of these peptides for eIF4E were found to be in the low nanomolar range similar to the affinity of the parent sTIP-05 peptide (Table 1). Significantly though, their rates of dissociation decreased relative to sTIP-05 and hence residence times were improved (68 seconds for sTIP-09 and 39 seconds for sTIP-10). The association rates were also lower for both sTIP-09 and sTIP-10 as compared to sTIP-05 (Table 1). These observations further substantiate the influence of the extended C-terminus on the binding kinetics.

Crystallization of the complexes of sTIP-09 and sTIP-10 with eIF4E (Fig. S3D, E and Table S1†) showed that the binding modes and interactions of these two peptides within the 12mer region were the same as in the parent molecule (Fig. 2D and E). However, in the sTIP-09 peptide, only R13 of the “^13^RRR^15^” sequence was completely resolved in the structure (Fig. S3D† and 3D). The guanidinium group of R13 was involved in cation–pi interactions with residues W73, Y76 and hydrogen-bond interaction with the side-chain of N77. For R14, only the backbone atoms were visible while no clear density was seen for R15. The crystal structure of sTIP-10 with eIF4E showed that the helical content of this peptide was relatively higher than in the other peptides because the *i*, *i* + 4 hydrocarbon staple stabilized an additional helical turn in the extended C-terminus of the peptide (Fig. 3E). However, this staple linker was still exposed to the solvent as observed in the other crystal structures, with no interactions made with the protein. The side-chain of Q13 behaved similar to W13 in sTIP-07 and R13 in sTIP-09, forming hydrogen-bond interactions with N77 and hydrophobic interactions with residues W73, Y76 and L131. Residue R14, being exposed to the solvent, did not form any specific interactions with eIF4E but should contribute to peptide solubility.

We computationally generated a complete model of the sTIP-09 peptide using the crystal structure as a template and subjected the complex to MD simulations (Movie S5†). The simulated trajectory showed that R13 was relatively stable, remained in contact with the protein residues (albeit *via* an unstable R13–N77 hydrogen-bond, occupancy < 10%) and hence, contributed favorably (–2.2 kcal mol^–1^) to binding along with the other core residues (Y4, R6, L9, L10 and F12; –1.7 to –7.3 kcal mol^–1^) in the peptide (Fig. 4D). R14 and R15 did not interact stably with the protein and this was also reflected in the absence of any significant energetic contributions from these residues (Fig. 4D). They were highly dynamic, corroborating the lack of density in the crystal structure, similar to that seen for the “ButPhI” functional group in &13. Simulations also showed that the extended poly arginine sequence formed a transient helical turn that fluctuated between random and ordered conformations (Movie S5†). MD simulations of the sTIP-10 and eIF4E crystal structure showed that the peptide maintained the additional helical turn observed at the C-terminus primarily because of the stability provided by the hydrocarbon linker (Movie S6†). Residue Q13 in the extended terminal segment contributed favorably (–2.0 kcal mol^–1^) to binding through its predominantly hydrophobic interactions with eIF4E along with the core residues (Y4, R6, L9, L10 and F12; –2.1 to –6.9 kcal mol^–1^) in the peptide (Fig. 4E). In summary, we observed that the C-terminal extension of the peptide engages the patch on eIF4E and provides additional new opportunities for stapling; the stapling also resulted in enhancing the helicity of the peptide and together, these modifications improved the residence time of the peptide–protein complex, while maintaining high affinity for eIF4E.

### Stapled-peptide with truncated N-terminus show moderate affinity for eIF4E

**Fig. 5:**
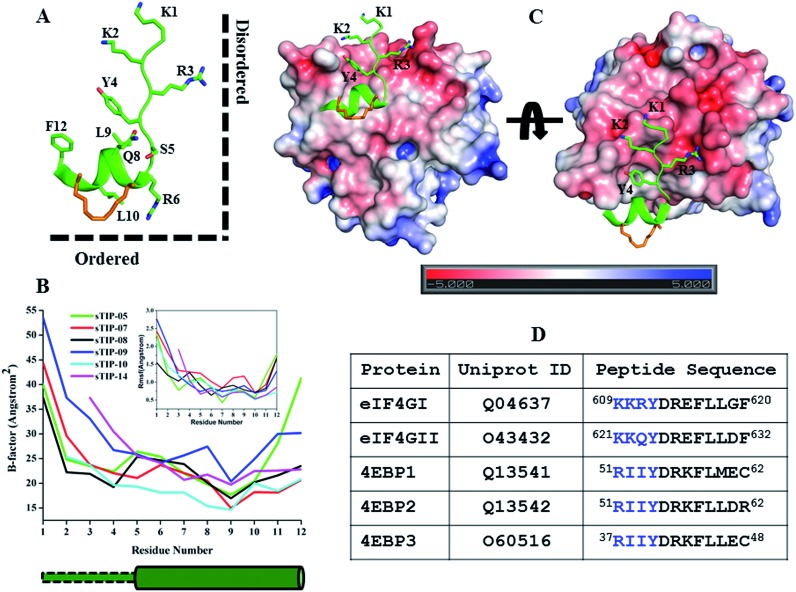
Physicochemical property of the N-terminal. (A) The canonical bound-state structure of the 12mer hydrocarbon stapled-peptide represented by sTIP-05. The disordered N-terminal region (absence of a defined secondary structure) and the ordered C-terminal segment are orthogonal to each other and highlighted to indicate the “Reverse L-shaped conformation”. (B) B-Factor and root mean square fluctuation (Rmsf) values of the CA atoms of the peptides. Only residues 1–12 across all the peptides are compared. The original B-factors values of sTIP-05 and sTIP-14 were respectively multiplied and divided by a factor of two for comparative analysis with other peptides. The rmsf (shown in inset) is computed with reference to the energy minimized structure of the respective peptides. The cylindrical and rectangular sketch below the plot represents the ordered helical and disordered random states respectively. (C) Crystal structure of sTIP-05 in complex with eIF4E. The protein is represented in electrostatic surface and the side-chain of the residues in the N-terminal region of the peptide are shown explicitly. The electrostatic potential surface was created using the APBS plugin through the PyMol molecular visualization software (Schrödinger). A colour gradient from blue to red represents the range of surface potential *kT*/*e* values from strongly positive (+5.0) to strongly negative (–5.0). (D) Isoforms of human eIF4G and 4EBP proteins, their Uniprot ID and the respective 12mer peptide segments that interact with eIF4E. The residues across these peptides that are structurally equivalent to the N-terminal region in sTIP-05 are emphasized in blue colour.

As observed in all the crystal structures, the bound-state conformation of the peptides had a disordered N-terminal region (absence of a defined secondary structure) and its orientation is almost orthogonal to the ordered C-terminal segment, resulting in a “Reverse L-shaped conformation” (Fig. 5A). The disordered N-terminal has a higher degree of flexibility, while the ordered helical region is relatively stable as can been seen from both the crystal (B-factor values) and simulated (Rmsf values) structures (Fig. 5B). The ^1^KKRY^4^ (or equivalent ^1^RIIY^4^) sequence in the disordered region of the peptide, does not form any specific intermolecular interaction with eIF4E across the different crystal structures except for the conserved tyrosine (Y4; hydrogen bond with P38) (Fig. 2). The chemical nature of the eIF4E surface around the N-terminal region of the peptide is predominantly electronegative while the region around the ordered segment is hydrophobic (Fig. 5C). This indicates that the major attractive force between the N-terminus of the peptides and the protein is a non-specific electrostatic interaction. A comparative analysis of the energetic contributions from the N-terminal residues (^1^KKR^3^ and ^1^RII^3^) across different stapled-peptide derivatives (Fig. 4A–E) showed that arginine, irrespective of its location in the sequence (R3 or R1) contributed significantly (≥–4.0 kcal mol^–1^) to the overall binding though with high deviations (Fig. 4A–E) reflective of an unstable intermolecular mode of interaction.

We systematically investigated the influence of this segment on the recognition of the peptide by sequentially deleting the “^1^KKR^3^” sequence from the 12mer sTIP-05 stapled-peptide derivative (Table 1). Deletion of K1 (sTIP-11) had little effect on binding, K1K2 deletion (sTIP-12) reduced the affinity several fold while the complete deletion of the ^1^KKR^3^ sequence (sTIP-13) resulted in almost complete abrogation of binding. These deletion experiments indicated that the presence of at-least one arginine residue in the peptide was critical for potent binding to eIF4E. Sequence comparison of equivalent peptide regions from different isoforms of eIF4G and 4EBP proteins that are known to interact with eIF4E, showed that these regions were highly variable except for the conservation of one basic charged residue (Fig. 5D). This collectively suggested that a single positively charged residue at the N-terminus is important to steer the peptide *via* long-range electrostatic forces towards the negative potential present near the protein-binding interface on eIF4E.

### C-Terminal tailoring recovers eIF4E binding of N-terminal truncated stapled-peptide

The 10mer sTIP-12 stapled-peptide had only moderate binding affinity (*K*_D_ of 397 nM) for eIF4E and thus we synthesized two derivatives of this peptide with their C-termini extended (Table 1) to investigate if the potential interaction with the patch can improve affinity. This included a 12mer peptide with “^11^FR^12^” addition and F10L substitution (sTIP-14), and a 13mer peptide with an *i*, *i* + 4 staple (sTIP-15) around the extended “^10^FQR^13^” sequence at the C-terminus. Phenylalanine and glutamine residues characterize distinct chemical properties which are complimentary to the targeted patch on eIF4E. It is interesting to observe that sTIP-14 and sTIP-15 respectively showed a 2-fold and a 7-fold improvement (*K*_D_ of 195 nM and 54 nM respectively) in binding eIF4E; the latter is clearly comparable to other good binders reported in this study (Table 1). The 2-fold improvement in the *K*_D_ of sTIP-14 over sTIP-12 is primarily due to a 2-fold reduction in its rate of dissociation (*k*_off_: 0.058 s^–1^ and 0.14 s^–1^ respectively). The more significant 7-fold improvement in the *K*_D_ of sTIP-15 over sTIP-12 is due to a 10-fold improvement in its rate of association (*k*_on_: 42 × 10^–5^ M^–1^ s^–1^ and 4 × 10^–5^ M^–1^ s^–1^ respectively). These data indicated that the addition at the C-terminus could significantly compensate for the loss of affinity due to N-terminal deletion by influencing the binding kinetics.

**Fig. 6:**
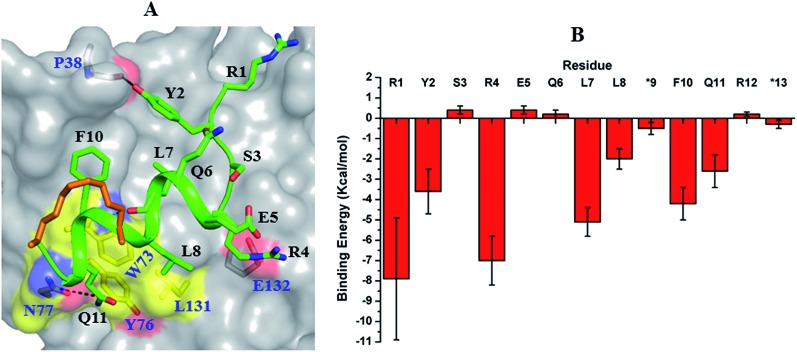
Modelled complex and residue-wise binding energy. (A) Modelled complex structure of sTIP-15 and eIF4E. The conserved canonical interactions, the new detected patch on the protein surface and its engagement by the C-terminal residue of the peptide are highlighted. Residue “R12” in the peptide is not shown for clarity. (B) The average binding energy and standard deviation of the hydrocarbon stapled-peptide residues computed from the ensemble of structures generated from MD simulations of sTIP-15 and eIF4E complex. The non-natural amino acids forming the hydrocarbon linker is represented by “*”. The computation was done using MM/GBSA method as described previously.^20^

The crystal structure of sTIP-14 peptide complexed with eIF4E was resolved (Fig. S3F and Table S1†) and showed that the peptide interacted in the canonical mode as observed for other peptides, albeit with a lesser degree of disorder at the N-terminus (Fig. 2F and 5B). This is the first structure of such a tailored eIF4E interacting stapled-peptide which physically demonstrates that despite the terminal modulation involving the deletion of the two lysine residues, the specific intermolecular interactions between peptide and protein are preserved. The phenylalanine residue (F11) of the added “^11^FR^12^” sequence efficiently interacts with the patch on eIF4E (Fig. 3F) by forming pi-stacking interactions with aromatic residues W73, Y76 and hydrophobic interactions with L131. The last arginine (R12) is exposed to the solvent and does not appear to form any specific interactions with the protein in the crystal structure. We also computationally modeled the complex-state structure of sTIP-15 peptide and eIF4E using the sTIP-10 structure as template since the extended C-terminus has an identical sequence and hence would likely form similar interactions with the protein (Fig. 6A).

Both complexes were subjected to MD simulations and the computed binding energy showed that F11 (–2.8 kcal mol^–1^) in sTIP-14 and Q11 (–2.6 kcal mol^–1^) in sTIP-15 make energetic contributions comparable to other critical residues (Y2, R4, L7, L8 and F10/L10; –2.0 to –7.0 kcal mol^–1^) in the peptides whereas R12 had only a negligible impact in both peptides (<–0.2 kcal mol^–1^; Fig. 4F and 6B). The sTIP-15 peptide had a significantly higher helical character as compared to sTIP-14 due to the additional helicity at the C-terminus. A comparative analysis of the simulated trajectories also showed that the C-terminus of the peptide is more stable in sTIP-15 (Movies S5 and S6†). Based on these observations, we speculate that increased helicity is associated with significantly higher rate of association for sTIP-15 compared to sTIP-14, and could be a primary factor for rendering it a more effective derivative in rescuing the N-terminal deletions.

## Discussion

A set of low nanomolar potent hydrocarbon stapled-peptide binders of eIF4E are described which are rationally designed and developed through structural insights into their molecular mechanism of recognition. Some of these peptide derivatives (sTIP-07 to sTIP-10) also exhibit improved residence time as compared to their parent (sTIP-05 and sTIP-06) compounds. These peptides with higher lifetime of complex formation have an extended C-terminus which was rationally designed to engage a specific local region on the surface of eIF4E. Residence time of binary drug–target complexes are increasingly being recognized as a key determinant for better pharmacological properties.^26^,^27^ The efficacy of a series of agonists of the adenosine A_2A_ receptor has been shown to be significantly correlated with their residence time.^28^ Antibacterial compounds that interact with the known antibiotic target LpxC with longer residence time are found to be more efficient in clearing bacterial infections in mice.^29^ Inhibitors of protein kinase p38, among others, were successfully optimized for improved binding kinetics during lead optimization and progressed to the clinical stage.^30^ These specific cases emphasize the advantage of considering both binding and kinetic properties in selecting lead molecules for further development towards preclinical pharmacological activity. Comparative structural and dynamic characterization of stapled-peptide: eIF4E complexes show that residues W13, &13, R13, Q13 and F13 in different peptides, despite differing chemical natures, are all consistently involved in stable hydrophobic interactions with the aromatic/hydrophobic (W73, Y76 and L131) residues that form part of the local binding area on eIF4E. In addition, a specific hydrogen-bond interaction between the side-chains of these residues and N77 (albeit with less stability) is also seen in all the complexes. These interactions result in significant binding energy contribution comparable to those from residues involved in canonical eIF4E recognition. The additional specific intermolecular interaction formed by the C-terminal extended stapled-peptides could be a primary factor that contributes towards lowering the rates of dissociation of these peptides from eIF4E. It should therefore be categorized as an important molecular determinant along with the other well-established canonical interactions in determining the optimum mode of complexation between stapled-peptides and eIF4E.

The rates of association are also observed to generally decrease as the peptide length is extended towards the C-terminus (for instance sTIP-07/sTIP-08 compared to sTIP-05/sTIP-06). One of the rate limiting steps in the association could arise from the degree of conformational rearrangement required to attain the bound state conformation. Molecular dynamics simulations of the free peptides in solution showed that the N-terminal segment is disordered whereas the C-terminal region which includes the stapled hydrocarbon linker largely adopts a helical conformation (Fig. S4A†). In the bound state too, the N-terminal segment remained disordered while the helicity of the C-terminal region increases and is observed to be more stable (Fig. S4B†). This indicates that these hydrocarbon stapled-peptides largely sample conformations that are predisposed for binding to eIF4E and they only undergo small reorganizational changes after docking with the protein. It is interesting to notice that the structural deviations between the bound and free conformations of the peptides are larger for sTIP-07/sTIP-08 as compared to sTIP-05/sTIP-06 (Fig. S4C and D†). This suggests that sTIP-07/sTIP-08 will have to undergo a relatively higher degree of reorganization to attain the bound state conformation compared to sTIP-05/sTIP-06. The formation of short-range intermolecular interactions with the protein could significantly impact the reorganization required. Hence, the additional specific interactions formed by the residues at the extended C-terminus (W13 and &13) for sTIP-07/sTIP-08 respectively could create a greater barrier and hence result in the lower association rates observed for these peptides as compared to sTIP-05/sTIP-06. However, there are also peptides which show a reduction in the rates of association due to deletion of residues from the N-terminal region (sTIP-12) or through a combination of both N-terminal deletion and C-terminal extension (sTIP-14, sTIP-15) as compared to the parent sTIP-05 peptide. The importance of the N-terminal segment is highlighted by the fact that sTIP-13 (deletion of K1K2R3) displayed negligible binding to eIF4E even though these three residues (K1K2R3) do not form any specific stable interactions with the protein other than likely steering the peptides towards the negatively charged surface near the protein-binding interface of eIF4E. So it is very probable that other factors such as the rate of diffusion (*via* electrostatic interactions) towards the binding interface also contribute to the differences in the observed rates of association.

**Fig. 7:**
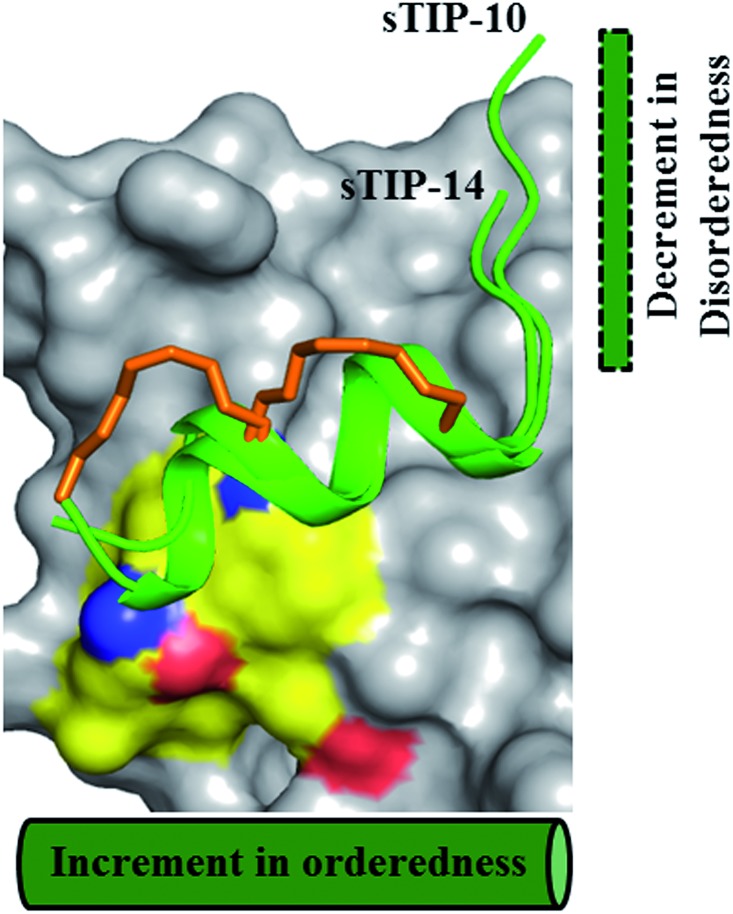
Terminal modulation and structural conformation of hydrocarbon stapled-peptide. Superimposition of the bound-state structures of sTIP-10 and sTIP-14 which highlight the distinct variations in the terminal regions of the peptide and the positions of the hydrocarbon linker. The backbone of K1 residue in sTIP-10 is modelled for comparison since it is not resolved in the crystal structure. The residues forming the patch on eIF4E are emphasized with a different colour combination as compared to the rest of the protein. The cylindrical and rectangular sketch represents the ordered helical and floppy disordered states respectively.

The bound-state conformations of sTIP-10 and sTIP-14 reveal that terminal modulation of their sequences results in interesting conformational properties of the peptides (Fig. 7). sTIP-14 was optimized with regard to the positive chemical potential at the N-terminal end which is as critical as the conserved “YXXXXLφ” motif for the peptide to recognize and interact with eIF4E. The outcome of this optimization was a decrease in the disordered state at the N-terminus of the peptide (Fig. 7 and 5B). sTIP-10, on the other hand, was developed in order to enable the peptide to engage the exposed region on the surface of the protein which also resulted in increased helicity towards the C-terminus. The combined outcome of this modulation was the evolution of a distinct class of hydrocarbon stapled-peptide compound (sTIP-15) against eIF4E that has a significantly reduced disorder at one end and increased helical order at the other (Fig. 6A). The reduction in the disordered N-terminal fragment serves as an excellent opportunity to develop peptides with better pharmacological properties as disordered flexible segments are prone to proteosomal degradation. The enhancement in the helical component alternatively would create ordered peptides with better stabilities. This could also aid in the cellular permeability of the peptides which is currently one of the major challenges in the development of stapled-peptide based compounds into mature therapeutic molecules.^31^ However, all these aspects need to be further explored in a cellular context to understand whether the improvement of the physicochemical properties of the stapled peptides translates into better cellular entry, stability, target engagement and activity. In summary, the findings from this work provide critical and new insights into the structure–activity relationship of hydrocarbon stapled-peptide interactions with eIF4E. This knowledge provided fresh avenues for development of these peptides, notably through their optimization of residence time, and hence offers the promise of evolving some of them into promising lead candidates for targeting eIF4E in oncology.

## Methods

### Chemical synthesis of hydrocarbon stapled-peptides

Fmoc solid-phase peptide synthesis was carried out in a 0.25 mmol scale on a Liberty Blue Automated Microwave Peptide Synthesizer (CEM) with Rink Amide MBHA resin (0.32 mmol g^–1^ loading). Olefin-bearing unnatural amino acids (*S*)-2-(4′-pentenyl) alanine or (*R*)-2-(4′-pentenyl) alanine were inserted at specified locations in the respective peptide sequences (sTIP-05 to sTIP-13, Table 1). All peptide couplings were performed under microwave irradiation (90 °C for 2 min.) with Fmoc-protected amino acids (5 equiv.) in DMF, DIC (0.5 M in DMF) and Oxyma Pure (1 M in DMF). Fmoc deprotection was achieved using 20% piperidine in DMF under microwave irradiation (90 °C, 1 min). Following final deprotection, *N*-acetylation was achieved using 10% acetic anhydride in DMF (10 ml) with heating (CEM Discover oven; 65 °C, 4 min). The resin was filtered and washed with DCM (×2). Under an inert atmosphere, first generation Grubbs catalyst (0.25 mmol) was added to the resin (0.25 mmol) and the glass vessel was sealed and well degassed before the addition of 1,2-dichloroethane (20 ml). The reaction mixture was degassed once again and left to stir at 23 °C for 18 h. The initial purple mixture became brown in the end and was filtered. The resin was washed with DCE (×2). A cleavage cocktail (10 ml) composed of TFA/thioanisol/EDT/anisole (90 : 5 : 3 : 2) was added to the resin in a BIOTAGE vial and the mixture was shaken for 3 h. The resin was filtered and diethylether (35 ml) was added to the peptide in solution and the mixture was centrifuged for 10 min. The diethylether solution was decanted and the precipitation/centrifugation step repeated twice. The crude peptide was dissolved in water + 0.2% TFA and purified by PREP HPLC to reach more than 90% purity.

### eIF4E protein expression and purification

The details on the cloning, expression and purification of eIF4E protein are described in the ESI.†

### Surface plasmon resonance

Human recombinant eIF4E protein with His tag (eIF4E-His) and without tag (eIF4E w/o tag) were separately immobilized on a CM5 sensor chip through amine coupling using the same protocol. Each flow cell of a CM5 sensor chip was first activated by a 7 min injection (10 μl min^–1^) of freshly prepared 1 : 1 50 mM NHS : 200 mM EDC. Diluted eIF4E in NaAc (pH 5.0), with m^7^GTP present in ≥2 : 1 ratio to saturate eIF4E, was injected over the sensor chip surface at a flow rate of 10 μl min^–1^. The remaining active coupling sites were blocked with a 7 min injection of 1 M ethanolamine at 10 μl min^–1^. The immobilization level is ∼2500 RU for eIF4E w/o tag and ∼5000 RU for eIF4E-His separately. Running buffer for immobilization was HBS-EP+ (10 mM HEPES, 150 mM NaCl, 3 mM EDTA, 0.05% surfactant P20).

Before measurement, the system was primed with assay running buffer HBS-EP+ (10 mM HEPES, 150 mM NaCl, 3 mM EDTA, 0.05% surfactant P20), with 1 mM DTT and 3% DMSO. Peptides were prepared by 3-fold dilution from high concentration to low concentration (3 μM to 1.372 nM). Peptides at increasing concentrations were injected over the chip surface for 60 s. The exposure was followed by a dissociation phase of 120 s. The flow rate was 30 μl min^–1^. Surface regeneration was done using 2 M NaCl 30 s at 30 μl min^–1^. Each reaction cycle ended with 50% DMSO extra wash. The solvent correction curve was setup by adding varying amounts of 100% DMSO to 1.03× running buffer to generate a range of DMSO solutions (2.000%, 2.286%, 2.571%, 2.857%, 3.143%, 3.429%, 3.714% and 4.000% respectively). After removing reference (blank buffer) signal and adding solvent correction, kinetics and/or steady-state parameters were calculated with Biacore T200 evaluation software ver. 3.0.

### Crystallization, structure determination and refinement

The crystallization of hydrocarbon stapled-peptide: eIF4E complexes, and the subsequent data collection and refinement were done at Novalix Pharma and Beryllium Discovery. The specific details on the experimental conditions for each complex structure are described in the ESI.† X-ray datasets were processed and scaled with the XDS^32^ and CCP4 (ref. 33) packages. The structures were solved by molecular replacement with the program PHASER^34^ using the human eIF4E structure from the PDB ID: ; 4TPW (chain A) as a search model. The starting models were built and refined by iterative cycles of manual and automatic building with Coot^35^ and restrained refinement with Refmac.^36^ For the eIF4E^HIS^: sTIP-09 complex, the molecular replacement model was PDB ID: ; 4BEA due to similarity of the unit cell and space group and the structure was refined in PHENIX.^37^ The geometric restraints for the non-natural amino acids constituting the hydrocarbon staple and the covalent bond linking their respective side chains together, to form the macrocyclic linkage constraining the sTIP peptides, were defined and generated using JLigand.^38^ Models were validated using RAMPAGE^39^ and the MOLPROBITY^40^ webserver. Final models were analysed using PyMol molecular visualization software (Schrödinger). See Table S1† for data collection, parameters and refinement statistics.

### Modeling and simulations

The hydrocarbon linker in the stapled-peptides and the modified amino acid “Lys(ButPhI)” were modeled using the XLEAP module of AMBER 14.^41^ Their partial charges (RESP) were obtained through the R.E.D server.^42^ Other force–field parameters were modelled using the all-atom ff99SB^43^ and GAFF^44^ force fields (AMBER 14). All *in silico* amino acid changes and modeling were performed using the PyMol molecular visualization software (Schrödinger). The atomic coordinates for residues 206–210 (numbering as per Uniprot ID: P06730) of eIF4E were not resolved in any of the crystal structures and hence were modeled using a previously determined crystal structure of eIF4E (PDB ID: ; 2W97) as template as it has resolved coordinates for this region. Any other unresolved side-chain atoms in the structures were modeled using the TLEAP module of AMBER 14. The terminal ends of eIF4E and stapled-peptides were capped with ACE and NHE functional groups respectively. The structures were solvated with TIP3P water^45^ in a cuboid box ensuring a minimum distance of 10 Å between the structure and the box boundary. The net charge of all the systems was positive and hence neutralized by adding the appropriate number of negative charges (chloride ions). The PMEMD module of AMBER 14 was used to carry out molecular dynamics simulations with a time step of 2 fs. Each system was first subjected to energy minimizations (using steepest descent and conjugate gradient algorithms) and then heated to 300 K with three different initial velocities under NVT conditions. They were then each equilibrated for 500 ps and subjected to 100 ns of production dynamics (totaling 8 different systems, each simulated in 3 replicates) in the NPT ensemble; the cumulative simulation time was 2.4 microseconds. The starting conformations for molecular dynamics simulations of the free peptides (sTIP-05, sTIP-06, sTIP-07, sTIP-08, sTIP-09, sTIP-10, sTIP-14 and sTIP-15) in solution were obtained from their respective bound state crystal/modeled structures. The solvation, system net charge neutralization, energy minimization, heating and equilibration were performed as described for the complex simulations above. Each of the peptides was simulated for a period of 500 ns production run in an NPT ensemble (totaling 4 microseconds for eight peptides). The regulation of simulation temperature (300 K) and pressure (1 atm), treatment of electrostatic interactions and constrain for bonds involving hydrogen atoms were implemented as previously described by Lama *et al.*^20^

### Data deposition

All the atomic coordinates of the crystal structures are deposited in the Protein Data Bank under the submission code 5ZJY (sTIP-05: eIF4E), ; 5ZJZ (sTIP-07: eIF4E), ; 5ZK9 (sTIP-08: eIF4E), ; 5ZML (sTIP-09: eIF4E), ; 5ZK5 (sTIP-10: eIF4E) and ; 5ZK7 (sTIP-14: eIF4E).

## Author contributions

S. A., C. S. V., C. J. B., T. B. and D. P. L. designed the project. A.-M. L. and S. A. performed stapled-peptide synthesis and SPR data analysis. Y. F. and J. N. performed eIF4E protein expression and purification. D. L., C. S. V. and N. T. performed molecular modeling and simulations. C. J. B. refined the crystallographic data and deposited the coordinates in the protein data bank. All the authors discussed the results and commented on the data. D. L. compiled, analyzed and organized the data for the manuscript. D. L. wrote the manuscript with editing from C. S. V and S. A. and proof reading from all the other co-authors.

## Conflicts of interest

Chandra S. Verma is the founder director of Sinopsee therapeutics, a biotech company developing molecules for therapeutic purposes; the current work has no conflict with the company.

## Supplementary Material

Supplementary information
