# Supplementary material for: Structural insights reveal a recognition feature for tailoring hydrocarbon stapled-peptides against the eukaryotic translation initiation factor 4E protein
Source: Chem Sci. 2019 Jan 7;10(8):2489–500. doi: 10.1039/c8sc03759k (PMC6385854; doi:10.1039/c8sc03759k)
Supplement: Supplementary file 1 [file SC-010-C8SC03759K-s001.pdf]

Electronic Supplementary Material (ESI) for Chemical Science.
This journal is © The Royal Society of Chemistry 2018

Supporting information

Structural insights reveal a recognition feature for tailoring hydrocarbon stapled-peptides against the eukaryotic translation initiation 4E factor protein

Dilraj Lama[1#], Anne-Marie Liberatore[2#], Yuri Frosi[3], Jessica Nakhle[2], Natia Tsomaia[4], Tarig Bashir[2], David P Lane[3], Christopher J Brown[3*], Chandra S Verma[1, 5, 6*] and Serge Auvin[2*]

[1]Bioinformatics Institute, A*STAR (Agency for Science, Technology and Research), 30 Biopolis Street, #07-01 Matrix, Singapore 138671

[2]Ipsen Innovation, 5, Avenue du Canada, Les Ulis - France 91940

[3]p53 Laboratory, A*STAR (Agency for Science, Technology and Research), 8A Biomedical Grove, #06-04/05, Neuros/Immunos, Singapore 138648

[4]Ipsen Bioscience, 650 East Kendall Street, Cambridge, MA 02142, United States

[5]Department of Biological Sciences, National University of Singapore, 14 Science Drive 4, Singapore 117543

[6]School of Biological Sciences, Nanyang Technological University, 50 Nanyang Drive, Singapore 637551

[#]Equal authorship

Address correspondence to:

Christopher J Brown
p53 Laboratory, A*STAR (Agency for Science, Technology and Research),
8A Biomedical Grove, #06-04/05, Neuros/Immunos, Singapore 138648
Tel: (65) 6478 8273
E-mail:cjbrown@p53lab.a-star.edu.sg

Chandra S Verma
Bioinformatics Institute, A*STAR (Agency for Science, Technology and Research),
30 Biopolis Street, #07-01 Matrix, Singapore 138671
Tel: (65) 6478 8273
E-mail: chandra@bii.a-star.edu.sg

Serge Auvin
Ipsen Innovation,
5, Avenue du Canada, Les Ulis - France 91940
Tel: (33)160 922481
E-mail: sergeauvin@gmail.com

Movies S1 to S8: Molecular dynamics simulation trajectories of stapled-peptide: eIF4E complexes. All the movies from the MD trajectories can be downloaded from http://web.bii.a-star.edu.sg/bmad/sTIP. The effective length of eIF4E protein considered for modeling and simulation is from residue 36-217 (numbering as per Uniprot ID: P06730). The protein is represented in ribbon (gray) and the residues (W73, Y76, N77 and L131) which form the patch on the protein binding interface are highlighted in surface depiction (yellow). The backbone of the peptide is in cartoon (green) and the side-chain of the residues along with the hydrocarbon linker (orange) is shown in stick representation.

Methods

eIF4E protein expression and purification

eIF4E (V28-V217, UniProt ID P06730) was produced in Escherichia coli (BL21 (DE3)) cells grown in LB medium. Bacteria from 3L of culture were harvested and lysed by sonication in buffer A containing 50mM Tris-HCl pH7.5, 100mM NaCl, Triton 1%, 5mM TCEP and 1 tablet of complete protease inhibitor. The lysed cells were centrifuged at 25,000 x rpm for 60min at 4°C. Soluble extract was passed through a diethylaminoethylcellulose (DEAE) column equilibrated in buffer A. The flow-through was diluted 5 times in 50mM Tris-HCl pH7.5, 0mM NaCl, 5mM TCEP buffer to decrease salt concentration to 20mM (so called buffer B). Diluted flow-through was then applied to a cation exchange chromatography (Hitrap® S HP, GE Healthcare) equilibrated in buffer B. A gradient (50 column volume (CV)) was applied from buffer B to equivalent buffer supplemented with 500mM NaCl. The eluted fractions containing protein were pooled and concentrated to 4ml to allow injection on gel filtration chromatography (Superdex® S75 16/60, GE Healthcare) in final buffer Na-Hepes 10mM, NaCl 125mM, TCEP 1mM. Fractions containing pure

protein were pooled and concentrated by ultra-filtration through a 10-kDa-cutoff centrifuge filter to 8.5mg/ml. TCEP was added to a final concentration of 10mM. Protein concentration was determined by Bradford assay. 1 liter culture yielded approximately 2 mg pure protein.

For sTIP-09: eIF4E system, a construct of human eIF4E (residues 27-217, UniProt ID P06730) as a fusion protein with an N-terminal His6 smt tag was produced in *E. coli* (TOP10). The protein was expressed overnight at 25 ˚C in 8L of TB medium which was supplemented with 0.1% L-arabinose. For purification, the pellet from 8L of media was suspended in a buffer (1:4 ratio of pellet to buffer) consisting of 25 mM Tris pH 8.0, 200 mM NaCl, 10 mM imidazole, 50 mM arginine, 0.5% glycerol, 0.02% CHAPS, 1 mM TCEP and lysed by a limited lysozyme treatment (in the presence of EDTA-free PI tablets) and by microfluidization at 15,000 psi, with 2 passes on ice. The lysate was clarified by centrifugation at 42,000 rpm for 30 minutes at 4 ˚C. The lysate was further purified by affinity chromatography using 3 x 5 ml HiTrap Ni Fast Flow Columns. After elution, the peak fractions were pooled and dialyzed in buffer A (25 mM Tris pH 8.0, 200 mM NaCl, 10 mM imidazole, 50 mM arginine, 1 mM TCEP, 0.25% glycerol) using a 10kDa MW cut-off dialysis tubbing, for 4 hours at 4 ˚C. 500 µl of Ulp1 protease (batch 65911) was added during the dialysis for tag cleavage. Tag cleavage was monitored by SDS-PAGE, and determined to be complete after 4 hours. To remove the cleaved His-Smt tag and Ulp1, the mixture was passed back over 2 x 5 ml HiTrap Ni Chelating Columns. The unbound material was concentrated to 15.4 mg/ml and further purified by SEC using a 320 ml Sephacryl S200 equilibrated with a running buffer of 25 mM Tris pH 8.0, 200 mM NaCl, 1% glycerol, 1 mM TCEP. Peak fractions were pooled and concentrated to 19.5 mg/ml in SEC running buffer. The material was aliquoted into 100 µl aliquots, flash frozen in

liquid nitrogen and stored at -80 ˚C, with a final yield of 135.55 mg of highly purified protein.

Crystallization and data collection

sTIP-05: eIF4E

eIF4E was concentrated to 8.5 mg/ml and incubated with 3 molar equivalents of sTIP-05 peptide and 1 molar equivalent of m^7GTP for 2 hours at 4 °C. Sitting drops were prepared using a Nanodrop® robot. Co-crystals were obtained within 2 weeks at 22 °C under the following condition: 30% (w/v) PEG 6000 and 0.1 M Na-Hepes pH 6.5. eIF4E complex crystals were frozen in an equivalent mother liquor solution containing 20% (v/v) glycerol and then flash frozen in liquid nitrogen. X-ray diffraction was collected at the PROXIMA-2 beamline at the Soleil synchrotron (Fra). See Table S1 for data collection statistics.

sTIP-07: eIF4E

eIF4E was concentrated to 8 mg/ml and incubated with 3 molar equivalents of sTIP-07 peptide and 1 molar equivalent of m^7GTP for 2 hours at 4 °C. Sitting drops were prepared using a Nanodrop® robot. Co-crystals were obtained within 2 weeks at 22 °C under the following condition: 12.5% (w/v) PEG 20,000, 20% (w/v) PEG MME and 0.1 M MES/imidazole pH 6.5. eIF4E complex crystals were frozen in an equivalent mother liquor solution containing 25% (v/v) glycerol and then flash frozen in liquid nitrogen. X-ray diffraction was collected at the PROXIMA-1 beamline at the Soleil synchrotron (Fra). See Table S1 for data collection statistics.

sTIP-08: eIF4E

eIF4E was concentrated to 8 mg/ml and incubated with 3 molar equivalents of sTIP-08 peptide and 1 molar equivalent of m^7GTP for 2 hours at 4 °C. Sitting drops were prepared using a Nanodrop® robot. Co-crystals were obtained within 2 weeks at 22

°C under the following condition: 12.5% (w/v) PEG 1000, 12.5 % (w/v) PEG3350, 12.5% (w/v) MPD and 0.1 M MES/imidazole pH 6.5. eIF4E complex crystals were frozen in an equivalent mother liquor solution containing 20% (v/v) glycerol and then flash frozen in liquid nitrogen. X-ray diffraction was collected at the ID-29 beamline at the ESRF synchrotron (Fra). See Table S1 for data collection statistics.

sTIP-09: eIF4E^{HIS}

For complex formation, eIF4E^{HIS} was diluted to a concentration of 50µM in the following buffer: 25 mM Tris pH 8.0, 200 mM NaCl, 1% glycerol, 1 mM TCEP. This was then then combined with 200 µM of sTIP-09 to give a peptide to protein ratio of 4:1. The complex was allowed to incubate on ice for 1 hour followed by concentration. The final protein concentration for crystallization was 9.75 mg/ml. Crystals of eIF4E:sTIP-09 were obtained using a ratio of 2:1 of protein to well solution using the following crystallization conditions: 40% v/v Ethylene glycol, 20 % w/v PEG 8000, 0.3M Sodium nitrate, 0.3 Sodium phosphate dibasic, 0.3M Ammonium sulphate, 100 mM Sodium HEPES/MOPS (acid) pH 7.5 (Morpheus, C06). Crystallization was performed using the sitting drop format using MRC plates. Plates were prepared using the NT8 robot (Formulatix, Ltd). eIF4E complex crystals were flash frozen in liquid nitrogen using well solution as a cryoprotectant. X-ray diffraction data were collected at the APS beamline 21-1D-F. See Table S1 for data collection statistics.

sTIP-10: eIF4E

eIF4E was concentrated to 8 mg/ml and incubated with 3 molar equivalents of sTIP-10 peptide and 1 molar equivalent of m^{7}GTP for 2 hours at 4 °C. Sitting drops were prepared using a Nanodrop® robot. Co-crystals were obtained within 2 weeks at 22 °C under the following condition: 30% w/v PEG 6000 and 0.1 M Na-Hepes pH 6.5.

eIF4E complex crystals were frozen in an equivalent mother liquor solution containing 20% (v/v) glycerol and then flash frozen in liquid nitrogen. X-ray diffraction was collected at the ID-29 beamline at the ESRF synchrotron (Fra). See Table S1 for data collection statistics

sTIP-14: eIF4E

eIF4E was concentrated to 8 mg/ml and incubated with 3 molar equivalents of sTIP-14 peptide and 1 molar equivalent of m^7GTP for 2 hours at 4 °C. Sitting drops were prepared using a Nanodrop® robot. Co-crystals were obtained within 2 weeks at 22 °C under the following condition: 25% w/v PEG 4000, 0.2M potassium iodide and 0.1 M MES/imidazole pH 6.5. eIF4E complex crystals were frozen in an equivalent mother liquor solution containing 25% (v/v) glycerol and then flash frozen in liquid nitrogen. X-ray diffraction was collected at the PROXIMA-1 beamline at the Soleil synchrotron (Fra). See Table S1 for data collection statistics.

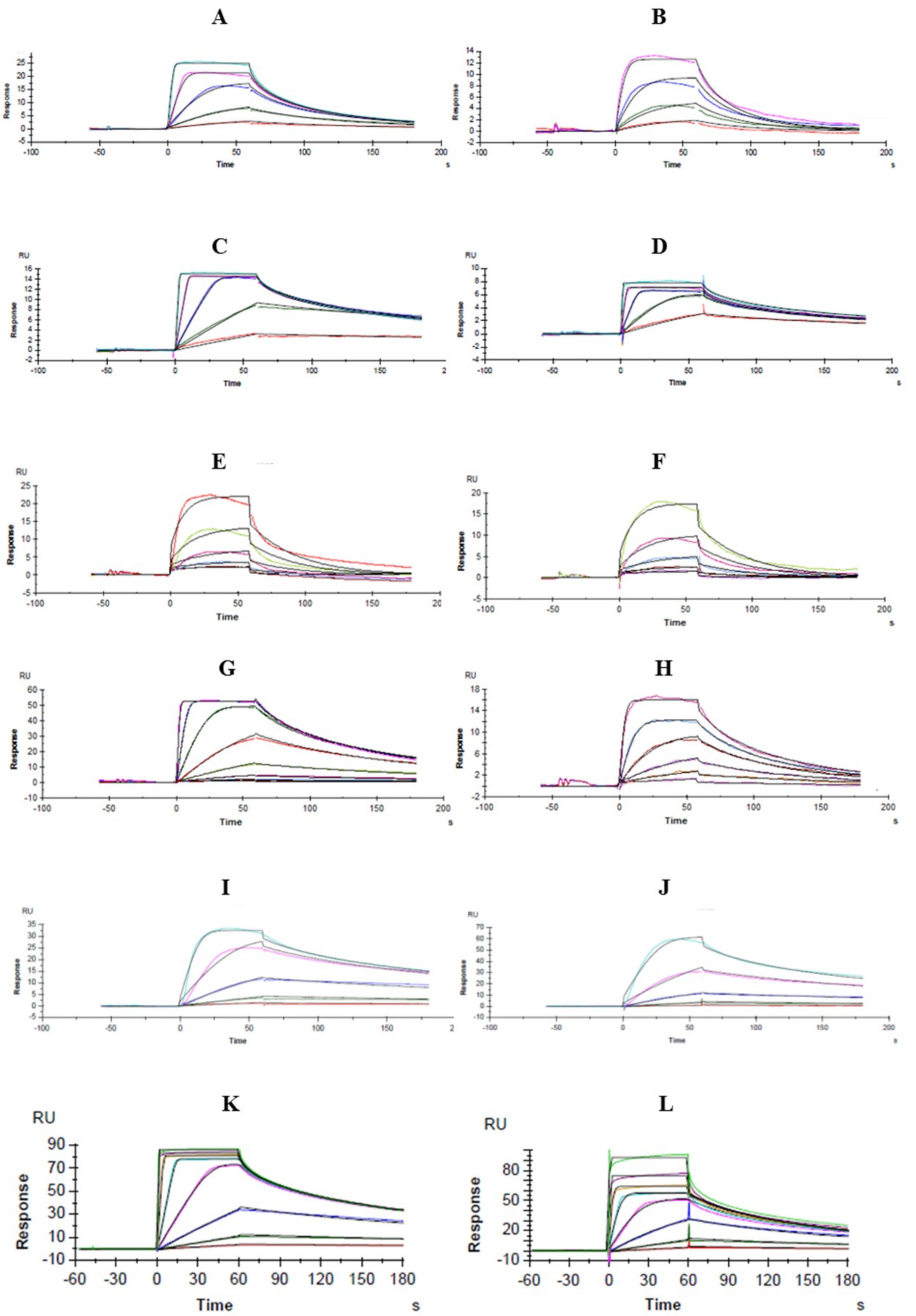

Figure S1: Sensograms. Two sensogram traces each for **(A, B)** sTIP-05, **(C, D)** sTIP-06, **(E, F)** sTIP-07, **(G, H)** sTIP-08, **(I, J)** sTIP-09 and **(K, L)** sTIP-10 hydrocarbon stapled-peptides obtained from two independent surface plasmon resonance (SPR) experiments.

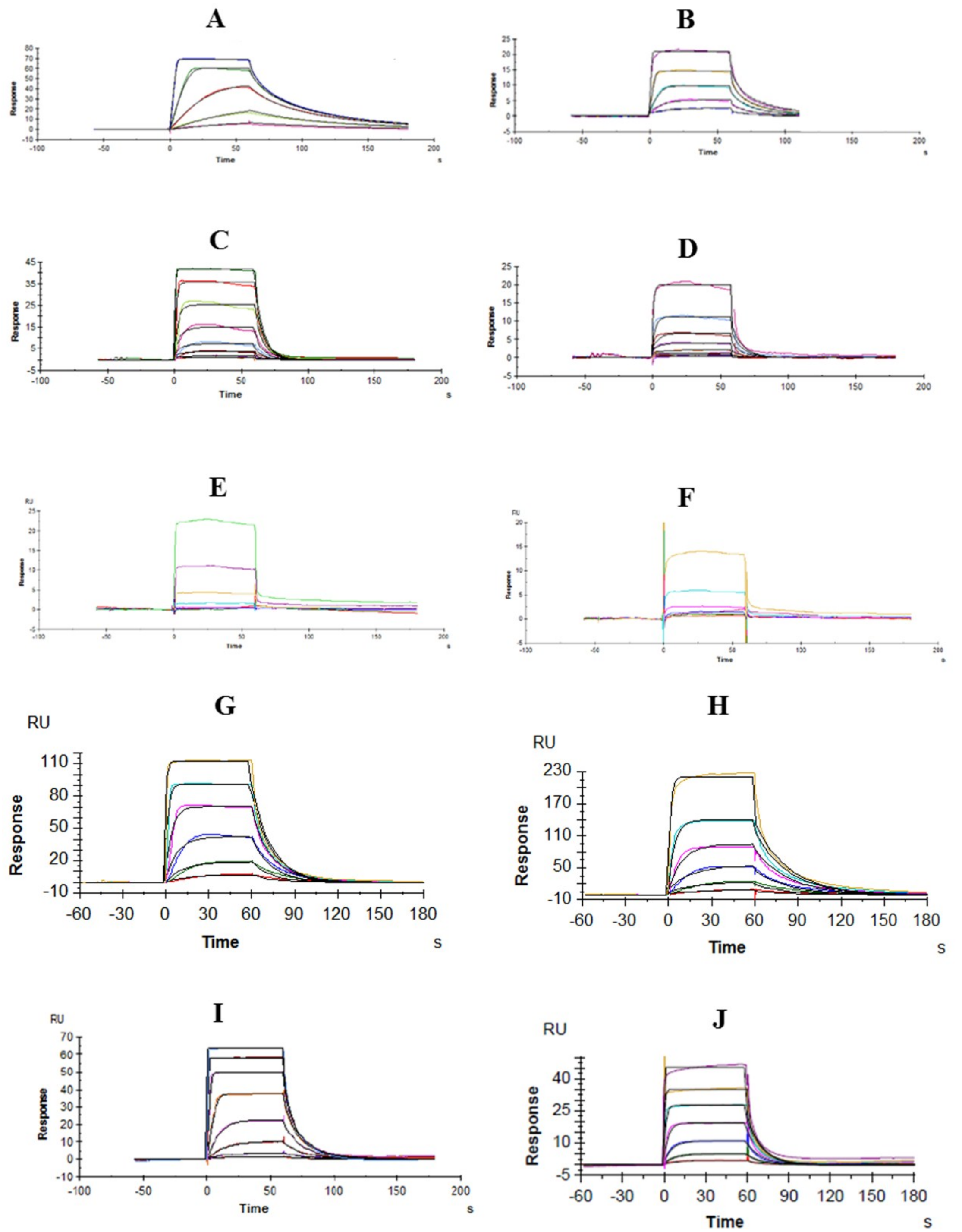

Figure S2: Sensograms. Two sensogram traces each for **(A, B)** sTIP-11, **(C, D)** sTIP-12, **(E, F)** sTIP-13, **(G, H)** sTIP-14 and **(I, J)** sTIP-15 hydrocarbon stapled-peptides obtained from two independent surface plasmon resonance (SPR) experiments. The binding affinity estimated for sTIP-13 was found to be 4μM and 36μM respectively.

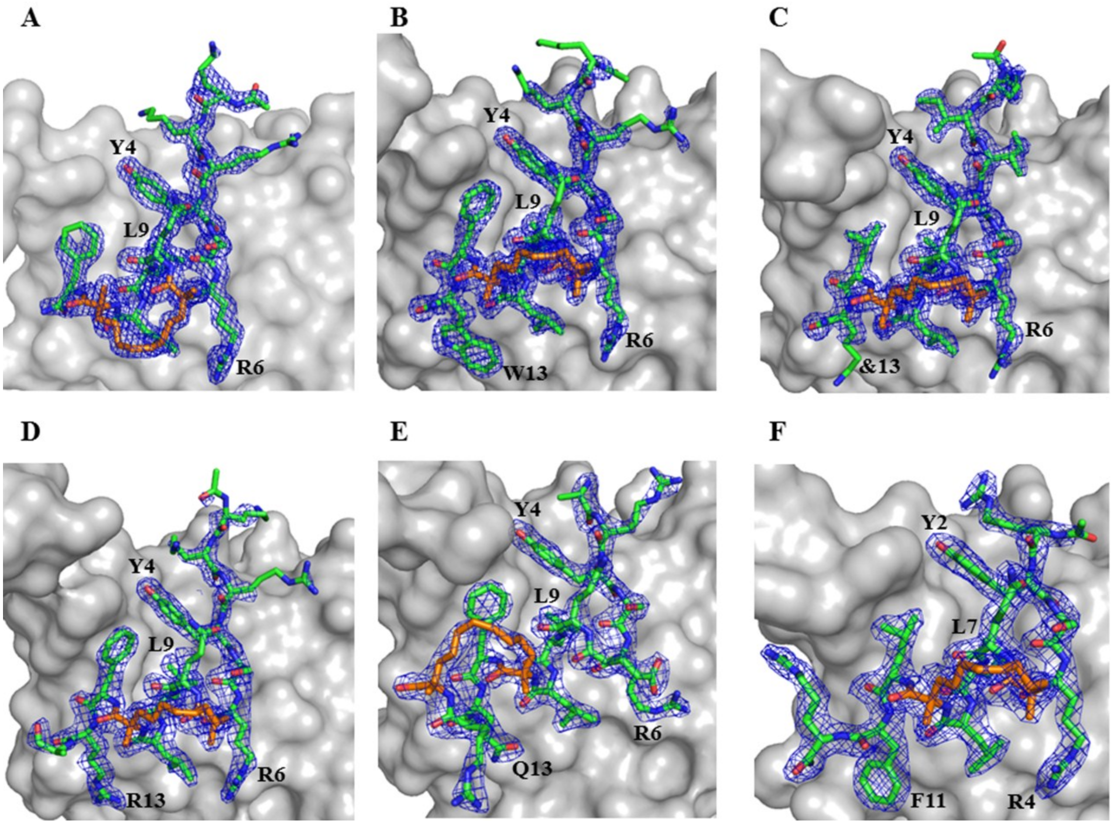

Figure S3: Crystal structures of hydrocarbon stapled-peptide: eIF4E complexes. **(A)** sTIP-05 (PDB ID: 5ZJY), **(B)** sTIP-07 (PDB ID: 5ZJZ), **(C)** sTIP-08 (PDB ID: 5ZK9), **(D)** sTIP-09 (PDB ID: 5ZML), **(E)** sTIP-10 (PDB ID: 5ZK5) and **(F)** sTIP-14 (PDB ID: 5ZK7)**.** The peptide is shown in stick representation, carbon atoms colored in green for all the residues except for the hydrocarbon linker which is colored in orange. The eIF4E protein is shown in surface representation in gray color. The backbone stereochemistry of the hydrocarbon linker in sTIP-05 is (R, R) whereas all the other peptides are in the (S, S) configuration. The 2Fo-Fc electron density map (blue mesh) demarcating the presence of the stapled peptides bound to eIF4E is shown at 1.5 sigma. The backbone stereochemistry of the hydrocarbon linker in sTIP-05 is (R, R) whereas all the other peptides are in the (S, S) configuration. Only representative residues from the peptide are labelled and the residue numbering is done as indicated in Table 1.

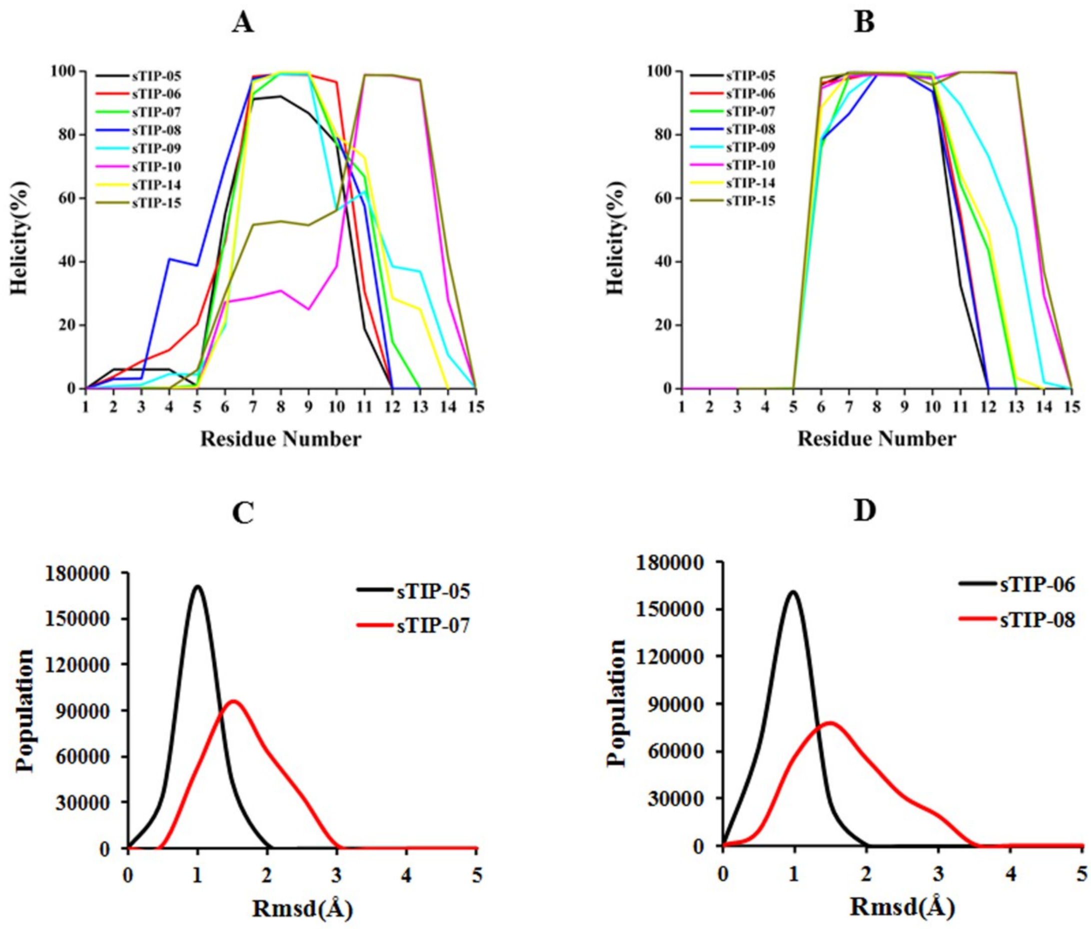

Figure S4: Residue helicity and structural deviation. Percentage helicity of stapled-peptide residues computed from molecular dynamics simulation of the peptides in **(A)** free and **(B)** bound states. The secondary structure was analyzed by using the "secstruct" command from the ptraj module of AMBER14. The reported percentage helicity is the summation of "3_{10}helix" and "alpha helix" values of the individual residues. **(C, D)** Root mean square deviation (Rmsd) computed between the ensembles of structures generated from molecular dynamics simulations of free and bound states of the stapled-peptides. Rmsd was computed by taking the main chain heavy atoms (N, CA, C and O) from the C-terminal region of the peptides (residues 6-12 for sTIP-05/sTIP-06 and 6-13 for sTIP-07/sTIP-08).

Table S1: Crystallographic data collection and refinement statistics.

Stapled-peptide: protein (PDB ID)	sTIP-05: eIF4E (5ZJY)	sTIP-07: eIF4E (5ZJZ)	sTIP-08: eIF4E (5ZK9)	sTIP-09: eIF4E (5ZML)	sTIP-10: eIF4E (5ZK5)	sTIP-14: eIF4E (5ZK7)
Resolution (Å)	50.00 - 1.59 (1.68 - 1.59)	50.00 - 1.67 (1.78-1.67)	50.00 - 1.76 (1.86 - 1.76)	50.00 - 1.80 (1.85 - 1.80)	50.00 - 2.25 (2.39- 2.25)	50 - 2.12 (2.25 - 2.12)
Space group	$P3_1$	C2	C2	$C222_1$	$P2_12_12_1$	$P2_12_12_1$
Unit cell dimensions (Å) and angles (°)	a=b=39.8, c=110.2 (α=β=90, γ=120)	a=91.3, b=38.3, c=78.9 (α=γ=90, β=122)	a=91.4, b=38.1, c = 78.5 (α=γ=90, β=122)	a = 91.4, b=38.1, c = 78.5 (α=β=γ=90)	a=37.5, b=65.1, c=76.4 (α=β=γ=90)	a=57.4, b=68.2, c=111.0 (α=β=γ=90)
Temperature (K)	100	100	100	100	100	100
Redundancy	5.0	5.7	5.8	6.1	8.7	9.4
Unique collected reflections	26096	26102	22709	22468	9319	25155
Completeness (%)	94.7 (99.1)	97.6 (94.4)	98.6 (97.1)	98.3 (95.7)	99.4 (99.0)	99.8 (98.9)
R Sym (%)	4.5 (59.2)	5.7 (62.8)	4.8 (44.1)	13.05 (3.17)	7.9 (46.4)	8.6 (61.7)
I/sigma	17.56 (2.08)	14.28 (2.26)	15.33 (2.18)	9.5 (49.7)	16.11 (3.13)	13.49 (2.70)
R factor (%)	17.83	18.95	17.71	18.47	19.83	21.55
R free (%)	21.60	21.21	21.15	22.26	26.84	26.99
RMS bonds (Å)	0.010	0.007	0.009	0.010	0.010	0.007
RMS angles (°)	1.74	1.50	1.68	1.42	1.75	1.35
Wilson B factor ($Å^2$)	22.1	13.6	14.2	16.3	27.7	28.6
Average B factors ($Å^2$)						
Chain A	29.01	19.89	22.60	25.95	37.47	42.54
Chain B	29.51	25.62	24.98	30.71	41.76	40.64
Chain C	NA	NA	NA	NA	NA	28.85
Chain D	NA	NA	NA	NA	NA	26.19
Waters	38.96	28.39	36.08	34.11	34.79	38.78
Number of water molecules	149	100	166	190	55	129
Ramachandran data (%)						
Favoured region	98.3	97.3	98.9	98.9	98.9	98.3
Allowed region	1.7	2.7	1.1	1.1	1.1	1.4
Outlier region	0.0	0.0	0.0	0.0	0.0	0.3

High resolution bin data stated in parentheses
